# Sensory Interactive Table (SIT)—Development of a Measurement Instrument to Support Healthy Eating in a Social Dining Setting

**DOI:** 10.3390/s20092636

**Published:** 2020-05-05

**Authors:** Juliet A. M. Haarman, Roelof A. J. de Vries, Emiel C. Harmsen, Hermie J. Hermens, Dirk K. J. Heylen

**Affiliations:** 1 Human Media Interaction, University of Twente, 7522 NB Enschede, The Netherlands; emielch@gmail.com (E.C.H.); d.k.j.heylen@utwente.nl (D.K.J.H.); 2 Biomedical Signals and Systems, University of Twente, 7522 NB Enschede, The Netherlands; r.a.j.devries@utwente.nl (R.A.J.d.V.); h.j.hermens@utwente.nl (H.J.H.)

**Keywords:** social dining, monitoring eating behavior, dietary support system, interactive technology

## Abstract

This paper presents the Sensory Interactive Table (SIT): an instrumented, interactive dining table. Through the use of load cells and LEDs that are embedded in the table surface, SIT allows us to study: (1) the eating behaviors of people in a social setting, (2) the social interactions around the eating behaviors of people in a social setting, and (3) the continuous cycle of feedback through LEDs on people’s eating behavior and their response to this feedback in real time, to ultimately create an effective dietary support system. This paper presents the hard- and software specifications of the system, and it shows the potential of the system to capture mass-related dimensions in real time and with high accuracy and spatial resolution.

## 1. Introduction

A balanced diet is essential for good health. The Global Burden of Disease study showed that a suboptimal diet is the second-leading risk factor for disability-adjusted life years and deaths worldwide [1]. However, the content of a balanced diet might vary from person to person, and even over the course of a lifetime within one person [2,3,4,5,6]. Finding and maintaining a balanced diet is a challenging task. Many forms of support exist that assist this process on an individual level. Traditionally, this form of support is done by dietitians that provide personal dietary advice and create awareness at the side of the individual by letting them collect insights about their eating behavior and habits [7,8]. Recently, this process of gathering insights is supported by technological developments such as smartphone apps [9,10], on-body wearables [11], instrumented dining trays [12], and cutlery [13] by collecting quantitative data about, for example, the exact amounts of micro and macro nutrients in the food, the quantity of the consumed food (per meal or per bite), or the speed with which a meal was eaten. Although these forms of support are all relevant when considering the individual aspects of eating, they do not take into account the social aspects of eating [14,15].

Many overt and covert dining interactions take place in these settings that can affect the adherence to personal dietary goals [16,17,18]. Passing on food during a celebration, notifying others about not eating specific food groups or using obtrusive technological measurement instruments in a communal setting are examples of overt actions that can increase the uncomfortableness at the side of the individual. Moreover, the numerous covert social interactions that take place during a meal unconsciously contribute to the food intake of an individual as well [19]. Synchronization of eating speed, feeling obligated to take food from a plate that is passed around, or adjusting overall dining time to table members are a few examples. Roughly 60–75 percent of the food that is consumed during a day is eaten in a social setting [20] and social events throughout life are often linked to food consumption. Therefore, solely targeting the individual diner independently in a quest towards a healthier diet does not seem to cover the full needs of the person.

As a first step in designing more effective dietary support systems for diners with health goals in a social setting, it is essential to gain more insights on: (1) the eating behaviors of diners in a social setting, and (2) the social interactions around the eating behaviors of diners in a social setting. Conventionally, (camera) observations are used to study social eating behavior [21,22,23,24]. Although this method provides valuable insights into multiple dimensions involved in social dining, such as the effects of conversation on the taste perception of a meal, eye contact between table members, or gesture mimicking, it misses out on many time-variant dimensions like the weight distribution of the food throughout the meal and per individual, or the quantity of the food that is shared with each other. Methods that do monitor these dimensions through technological attributes such as weighing scales or instrumented cutlery [12,13] highly intervene with the natural behavior of the table members and thereby act as a large confounder in this type of research. Alternatively, Chang et al. [25] introduced a dining table that automatically tracks what and how much people eat over the course of a meal, intended to track in-situ individual behavior in a natural social setting. Albeit the spatial resolution of the measurement instrument is very low, this development embodies a valuable step towards the creation of measurement instruments that help us in gaining new insights (such as food weight, food distribution, or bite size) on the behavior of an individual in a natural social dining setting. Moreover, measuring food related behaviors in this embedded way also creates the opportunity to give feedback on mass-related dimensions of eating around the table in real time. This new way of measuring creates the opportunity to study the continuous cycle of feedback and the response to this feedback in real time.

This paper presents the development of the Sensory Interactive Table (SIT): an instrumented, interactive dining table that is able to monitor individual eating behavior in a social setting. A dining table was specifically chosen as object to instrument, as it is the center piece of many natural social dining experiences and the dining surface offers numerous possibilities to, unobtrusively, store technology. The next sections explain the hard- and software that is embedded in SIT, the measurement features that can be derived from it, and it outlines the added value of these features in the context of social dining. In the last part, we discuss how these results relate to other academic standards that measure eating behavior.

## 2. Sensory Interactive Table (SIT)

**Figure 1:**
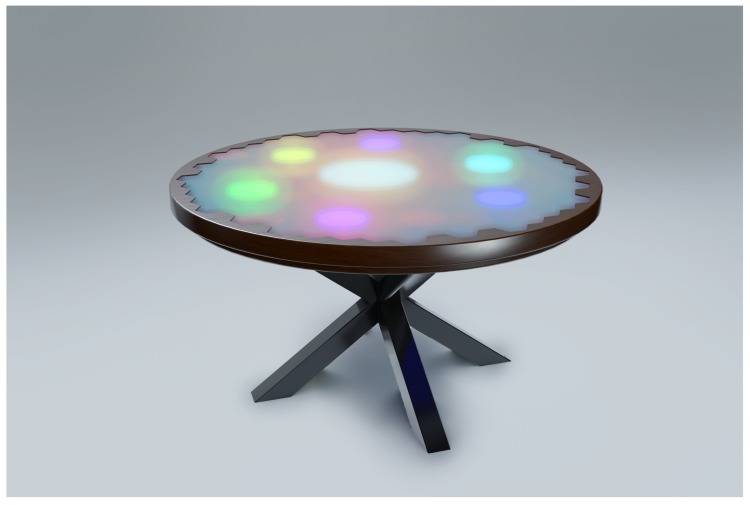
Sensory Interactive Table (SIT). Loadcells and LEDs are located below the tabletop surface, measuring the weight distribution of the items on the table and providing light interactions to the people at the table, respectively.

SIT is an instrumented, interactive dining table that is embedded with load cells and LEDs just below the tabletop surface. The load cells measure the weight of the items on the table, over the course of a meal. This way, many overt and covert aspects of eating behavior related to mass become measurable, such as bite size, total amount of food on a plate, or synchronicity of eating speed between table members. Simultaneously, the LEDs (Figure 1) allow for communication with table members by use of light interactions, potentially providing them with feedback and advice about their behavior, habits and eating choices, which is outside the scope of this paper.

### 2.1. Hardware

**Figure 2:**
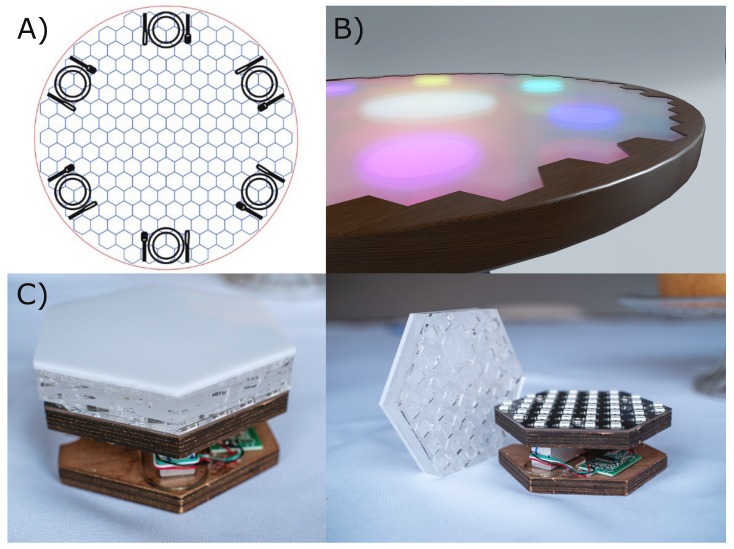
Composition of the sensors embedded in SIT. (**A**) spatial distribution of 199 modules over the table surface; (**B**) wooden outer layer of SIT does not comprise any sensors; (**C**) single module with loadcell, LED panel, and plexiglass diffusor on the top.

SIT is a round dining table (diameter 1.45 m) that can seat six people. The table surface is composed of 199 individually-controllable, hexagon-shaped modules. Figure 2A shows the configuration of the modules over the area of the table. The wooden outer layer (Figure 2B) of the table does not hold any sensors. Each module has a surface area of 7.6 × 10^3^ mm^2^ (side length 54 mm), and contains: (1) one load cell (HT Sensor Technology CO., Ltd., Xi’an, China) with a maximum loading capacity of 5 kg, (2) a HX711 24-Bit Analog-to-Digital Converter (ADC) (Avia Semiconductor, Xiamen, Ltd., Xiamen, China) that amplifies the voltage signal from the load cell and digitizes it into a 24-bit value, and (3) a custom made LED panel that contains 42 SK6812 digital RGB LEDs placed at a distance of 13 mm from each other (Figure 2C). The module size is chosen such that it allows for high spatial resolution of the measurement surface. The load cells and LEDs can be controlled both individually and simultaneously.

Each module is covered with a hexagon-shaped 15 mm-thick opaque plexiglass aimed to diffuse the LED light (Figure 2C). The thickness and material were chosen such that they match the level of diffuseness that is envisioned for the aimed research setting. Moreover, a small space was left between each module to obstruct the transmission of forces to nearby loadcells. A plastic foil is placed on top of the plexiglass to create a waterproof surface. A table cloth makes up the last layer of the table to create a visually appealing unobtrusive measurement instrument. The flexibility and thickness of both layers are chosen such that they do not physically obstruct the transmission of forces to the loadcells.

### 2.2. Software and Electrical Design

Unity (Unity Technologies) is a cross-platform game engine that is currently chosen to collect and process the data from the loadcells (input) and control the interactions that is sent back towards the LEDs (output). The software allows for individual processing of input and output alone, or can interconnect the two, creating a feedback loop to the user. It creates a flexible set-up, suitable to study the eating behaviors of people in a social setting, the social interactions between people in a dining setting, and the continuous cycle of feedback and the response to this feedback in real time.

All digitized loadcell data that are coming from the individual HX711 Load Cell Amplifiers are simultaneously collected at the table by three separate microcontrollers (ATMega2560, Sherwood, OR, USA). Each ATMega2560 covers the signals of 1/3 of the total amount of load cells that are embedded in the table. The data received by each of the three microcontrollers are fused by an ARM Cortex-M7 processor which operates at 600 MHz (Teensy 4.0, Zhuhai, China). The resulting sample frequency of the measurement system is 72 Hz. Additionally, based on the setting in which the table is used, filtering can be performed at the Teensy 4.0 microcontroller to remove spikes or to smoothen the signal for feedback by the LEDs. However, no filtering at the microcontroller was used in this paper as raw data was preferred for the data analysis.

Using serial connection, Teensy 4.0 sends the data to the Unity engine which can subsequently map the 24-bit integer value to a specific colormap. This is then sent back to the table, using a serial connection, to a 180 MHz Cortex-M4F (Teensy 3.6) which communicates with the LED matrix. The total delay between putting a mass on the load cell and providing visual feedback is currently 17 ms but logically depends on factors such as filter settings, the maximum frame rate of the PC, or the processing speed of Teensy 3.6.

## 3. Measurement Features

As stated, many levels of time-variant mass-related interactions take place in a social dining setting, such as the weight distribution of the food throughout the meal or the quantity of the food that is shared with each other. SIT should therefore be able to detect a large variety of features, all with their specific measurement requirements. Some features require highly accurate measurements, such as detecting individual bite sizes, eating speed, eating synchronicity of table members, or determining the total amount of food consumed by individuals. Other features require a set-up where the focus lies more on the spatial mapping of the eating or dining events: which foods are eaten first, or are left untouched; what is the placement of dining attributes, such as plates, glasses, or pots on the dining table; and how is food shared among table members. SIT should be able to both measure with high accuracy on an individual module level, as well as interconnect all individual signals to each other. In Section 3.1, Section 3.2, Section 3.3 and Section 3.4, we will explain in more detail how these features are implemented in our measurement instrument.

### 3.1. Measurement Accuracy of One Single Module

**Figure 3:**
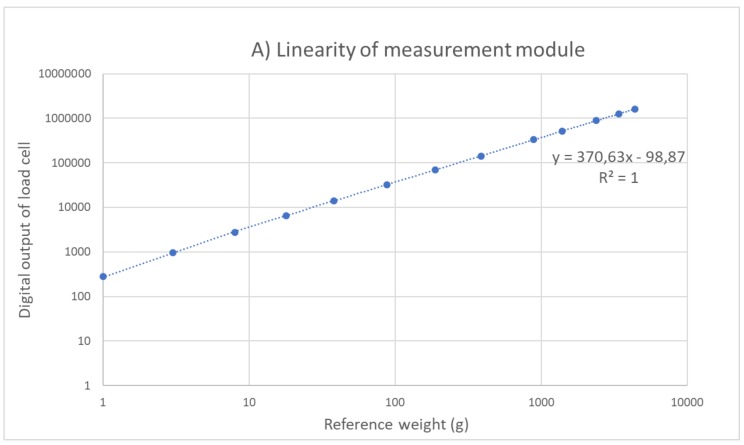

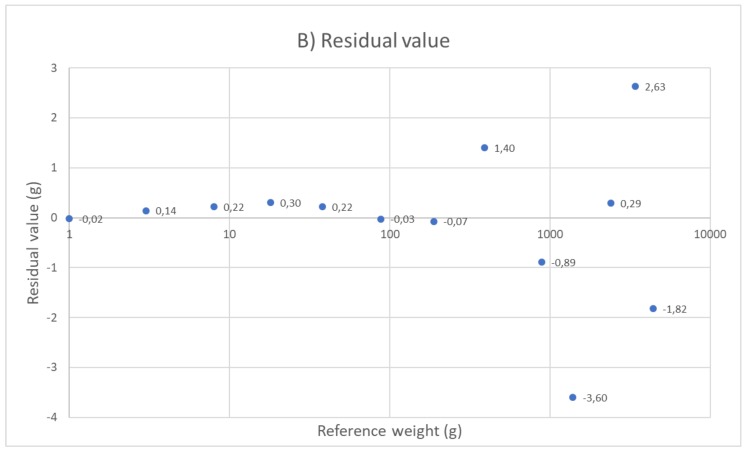
Sensor calibration procedure. (**A**) reference weight (range 1 g–4388 g) was plotted against digital output signal of the loadcell in order to determine the best fit on the data; (**B**) a linear fit was performed and found capable of producing realistic weight values. Residual values are plotted.

To determine the accuracy of our measurement instrument, the following calibration procedure on each of the individual modules in our system is needed. As a first step, sensor calibration was performed by incrementally putting reference weights on one individual module, ranging from one gram to 4388 g in steps of 1 g, 2 g, 5 g, 10 g, 20 g, 50 g, 100 g, 200 g, 500 g, 500 g, 1000 g, 1000 g, and another 1000 g. After putting the reference weight on the module, the signal was then recorded for one second and averaged over that time frame. The averaged values were plotted against the reference weight, to determine linearity of the sensor. By regression analysis, it was concluded that a linear fit was capable of producing realistic weight values (Figure 3A). The residual value between the reference weight and the regression fit is plotted in Figure 3B. The maximum residual value was equal to or less than 0.3 g (18 g reference weight in Figure 3B) in the range of 1 to 188 g (grams from 1 to 100 added up), whereas the maximum residual was equal to or less than 3.6 g (1388 g reference weight in Figure 3B) for weights up to 4388 g (all grams added up). Note that these values might change when different averaging windows are chosen.

**Figure 4:**
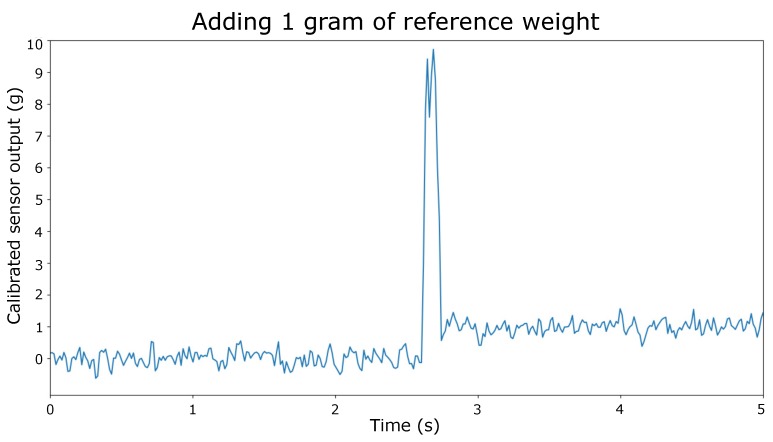
A reference weight of one gram was placed on top of the module. The large peak in the signal clearly indicates placement of the weight on the module.

Figure 4 shows the placement of a one gram reference weight on the module. Noise is visible around the mean value of 0 grams and 1 g, with an SD of 0.23 g. Although we could employ some form of low pass filtering to remove this noise, the figure also clearly shows the actual placement of the weight. Smoothing the signal reduces the amplitude of distinctive characteristics in the signal, whereas this peak value could be an important characteristic for event detection. This will be further discussed in Section 3.4. The implementation of a low-pass filter, and its specific filter settings might therefore depend on the intended use of the SIT and might vary between different use cases.

### 3.2. Combining Multiple Modules: Measuring Weight Distribution of a Small Surface

Dining attributes such as glasses, cutlery, plates, or pots are often larger than the size of one individual module (7.6 × 10^3^ mm^2^). Moreover, in a real-life scenario, module-sized attributes are often not positioned exactly on top of a single module. This section will cover the superposition of multiple modules and the fusion of data of these modules, to show the accuracy of SIT in doing so.

**Figure 5:**
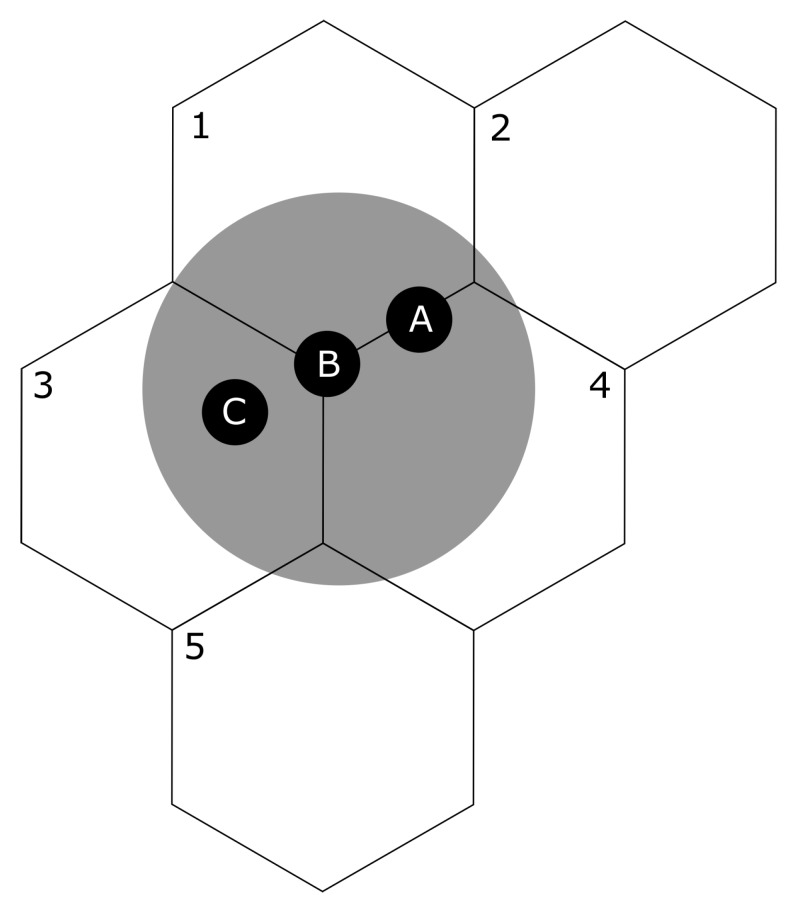
Measurement set-up: A dining plate was positioned over five modules, and a weight (500 g) was moved over the surface of this plate in three locations. Location A: the weight was positioned over modules 1 and 4. Location B: the weight was positioned over modules 1, 3, and 4. Location C: the weight was positioned over module 3.

As a use case, a glass dining plate was positioned over multiple modules and a weight (500 g) was moved over the surface of this plate throughout the measurement. Figure 5 shows the measurement set-up that was used. The weight was positioned at three different locations on the plate. Location A: the weight was positioned over modules 1 and 4. Location B: the weight was positioned over modules 1, 3, and 4. Location C: the weight was positioned over module 3. Loadcell data of all five modules were recorded continuously throughout this procedure and analyzed afterwards.

**Figure 6:**
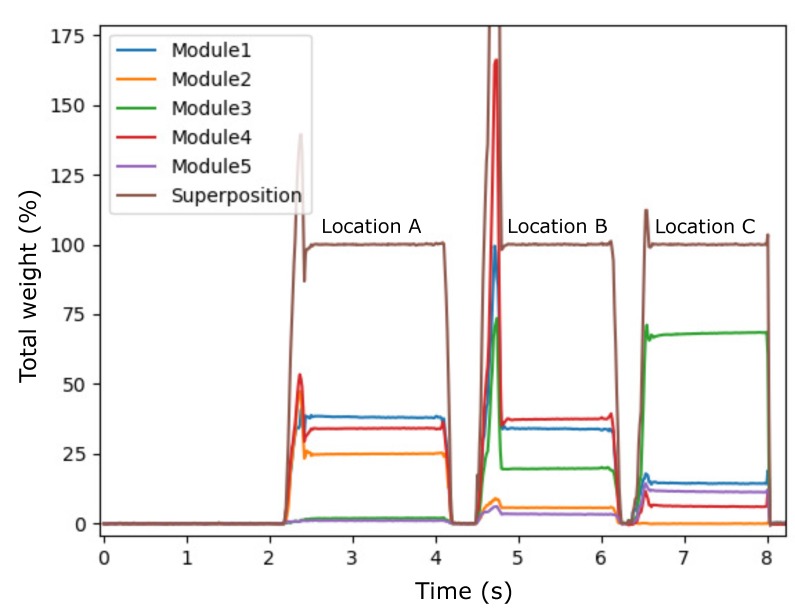
System response to the placement of the weight on three different locations on the dining plate (A, B, and C). For each module, loadcell data (expressed as percentage of total weight) are plotted individually over time. Superposition of the individual signals indicates that the total weight that is recorded by all individual modules equals 100 percent of the total weight that was used.

Figure 6 shows the result of this experiment. The duration of the experiment is plotted against the recorded weight on each of the individual modules (in percentage of total weight used). Additionally, the superposition of these signals is presented in the graph, for all three locations. For location A, signal response is the largest for modules 1 and 4, followed by module 2. As expected, little weight is recorded by modules 3 and 5, as these modules are furthest positioned from the location of the weight on the dining plate. The same counts for location B, where the largest signal response is recorded by Modules 1, 3 and 4 and the least response by modules 2 and 5. For location C, one can clearly see the large signal response of Module 3, and the decrease in response by the remaining modules.

The recordings fit the expectations that the system not only allows for continuous monitoring of the amount of food on a plate throughout the meal, but additionally allows for localization of the interactions with the food on the plate due to high spatial resolution of the measurement instrument. In a real-world use case, this means that we are confident that we will, for instance, be able to detect which foods on a plate are left untouched or which food is eaten first.

### 3.3. Spatial Representation of Dining Interactions

Information such as bite times, bite weights, and periods of chewing can already be extracted by solely looking at individual modules or small surfaces; however, one might miss out important dimensions of social dining when doing so. Examples of these dimensions are: the distribution of the food between the table members over the course of a meal; the relative amount of food table members serve themselves; and the order in which table members serve themselves [12,13]. To get a broader perspective on these interactions over the course of the meal, it is important to fuse individual loadcell data of the whole table.

**Figure 7:**
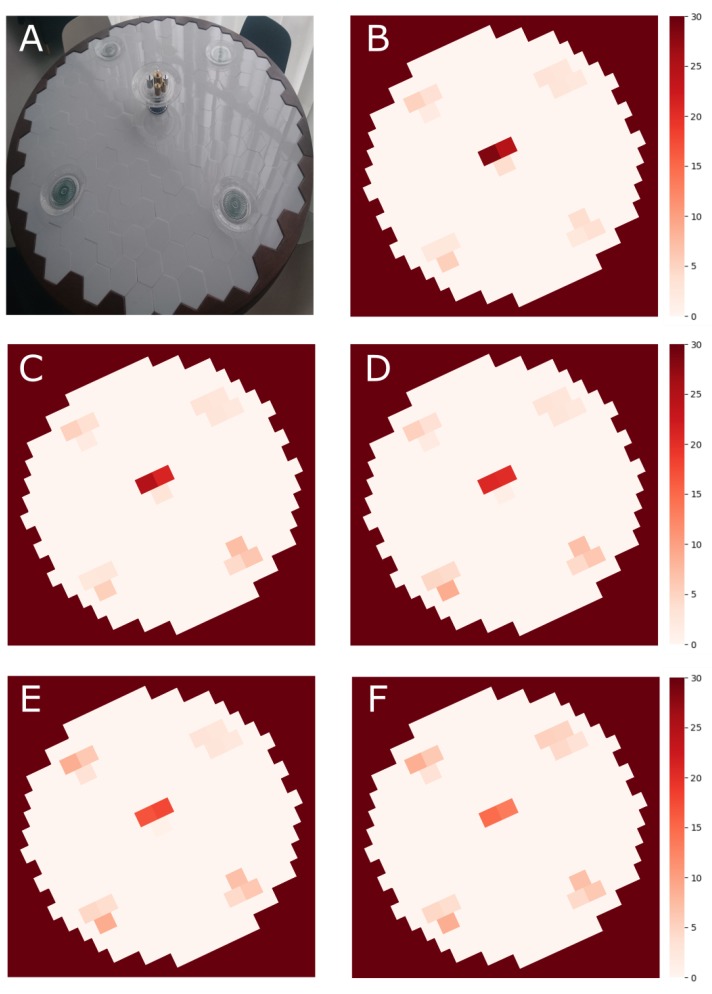
A set of five static heatmaps was created of a scenario where four table members share a pie which is served in the middle of the table. (**A**) reference picture of the starting scenario. The pie is served in the middle of the table. Plates are positioned at four seating locations of the table; (**B**) corresponding heatmap of the scenario presented in (**A**). (**C**–**F**) heatmap after table members one, two, three, and four put a piece of pie on their plate, respectively. At the end of the scenario (**F**), no pie is left on the serving tray in the middle of the table.

To visualize this perspective, a set of static heatmaps was created of a scenario where four table members take a piece of pie from a serving tray that was positioned in the middle of the table and placed it on their individual plate in front of them. To have accurate recordings, we symbolized the pie by several reference weights (in total 800 g) and distributed those over the dining plates (in the order of 250 g, 200 g, 200 g, and 150 g). The heatmap that is shown in Figure 7 shows the static situation at five moments in time during this experiment: first, at the start of the experiment where none of the subjects took a piece of pie and only the empty plates are visual on the heatmap (Figure 7A,B); second, after the first table member took a piece of pie on his plate (Figure 7C), and after the second, third, and fourth table member took a piece on their plate (Figure 7D–F, respectively).

The set of heatmaps not only visualizes the weight distribution of the food on the table throughout the process of sharing the pie; moreover, it also reveals interactions such as turn taking, the chosen portion size by each table member, the placement of the piece on their plate, and the process of waiting to eat until every table member has gotten a piece of food on his plate. In this specific scenario, turn taking was clockwise, and none of the table members started to eat before each person had a serving on his plate. At the end of the scenario, all pie was distributed over the four table members. The weight in the middle of the table reflects the weight of the serving tray.

### 3.4. Classification of Dining Related Events

Although the above measurement scenarios portray the potential of the measurement instrument, they are recorded under strict preset conditions. Analysis and interpretation of the data are therefore relatively straightforward. One can imagine that, during in-the-wild dining scenarios, many interactions take place at the same time, thereby making the measurement of interactions of interest more complicated. Dining related events can occur at all levels of the system: on a single module, over a small surface, or between table members. SIT should be able to classify these events by distinguishing characteristics in the raw data. The better the system is able to do so, the better the system is able to put the data in perspective.

**Figure 8:**
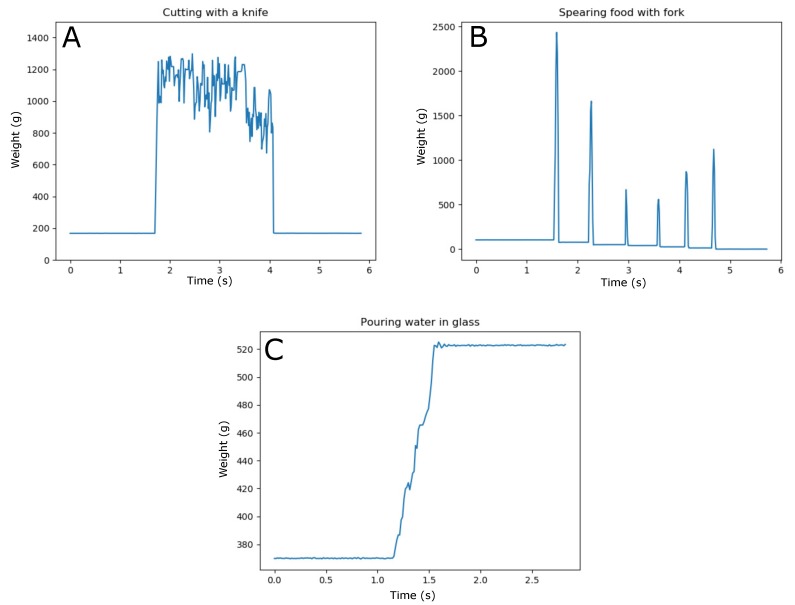
Three typical examples of dining related events have been recorded on a single module to show the differences in signal characteristics per event. (**A**) cutting food with a knife; (**B**) spearing food with a fork and taking it off the module; (**C**) pouring water in an empty glass.

To illustrate this process, we have identified typical examples of such events to demonstrate the types of events that can occur. Figure 8 shows three scenarios that have been performed on a single module: cutting food with a knife (Figure 8A); spearing food with a fork and taking it off the module (Figure 8B); and pouring water in an empty glass (Figure 8C). Even though the data are only recorded at the level of a single module, it clearly demonstrates the differences in signal response to the events that takes place. The process of finding typical characteristics to distinguish one event from another is a process that is still ongoing. Figure 8 serves solely as an example of this work. It demonstrates the high amount of information that is available in the raw data of one single loadcell, and shows the potential in defining many layers of interactions when combining multiple modules.

Scenario A clearly shows the increase and decrease of pressure on the module surface, thereby representing the back and forward movement of a knife while cutting a piece of food. The knife was moved in a continuous movement throughout this recording. No food was removed from the surface afterwards. Scenario B shows the peak loading that occurs while spearing pieces of food with a fork. The pieces of food were already pre-cut; therefore, no knife interaction is visible in this graph. The total weight of the food that was placed on the module decreases, after each instance of taking away a piece of food. Scenario C shows a gradual increase in weight, without any instances of peak loading. The total increase in weight corresponds to the capacity of a small drinking glass.

## 4. Discussion

Conventional forms of dietary support often target the individual eater when supporting dietary goals. These forms of support are all relevant when considering the individual aspects of eating; however, they do not take into account the social aspects of eating. Solely targeting the individual diner independently in a quest towards a healthier diet does not seem to cover the full needs of the person. In order to create more effective dietary support systems for people with health goals in a social setting, it is essential to develop systems that can measure (1) the eating behaviors of people in a social setting, (2) the social interactions around the eating behaviors of people in a social setting, and (3) the continuous cycle of feedback and the response to this feedback in real time. To this end, we developed the Sensory Interactive Table (SIT): an instrumented, interactive dining table. We presented the hard- and software specifications of the system, and the potential of the system to capture mass-related dimensions in real time and with high accuracy and spatial resolution.

### 4.1. Measurement Accuracy of One Single Module

As SIT should be able to identify eating behaviors of individuals in a social setting, we looked for literature standards as reference points for our system. Single bite size ranges between 5 (small bite sizes) and 15 g (large bite sizes), depending on the type of food that is consumed [26] and individual eating speed is found at 3 to 9 s between two consecutive bites [26]. Our system functions very well in light of these values. Measurement error of our system ranges between 0.3 g and 3.6 g, depending on the loading of a single module. Note that measurement errors have been determined on a signal that was averaged over one second. As stated, averaging data over a period of time reduces the noise in the signal, but could also lower the amplitude of signal characteristics that might be important for event classification. When designing filter settings, the time between two consecutive bites should be taken into account. This time can be seen as the lower limit at which the system should be able to process and filter data, and still be fast enough to provide valuable feedback to the user about this dimension. Our system currently operates with a processing time of 17 ms and therefore does not hinder the feedback in any way.

### 4.2. Combining Multiple Modules: Measuring Weight Distribution of A Small Surface

In a practical use case scenario, intake measurements are usually in relation to a food portion, positioned on a dining plate that covers multiple modules. As an example, we imagine a dining plate (500 g) with food (350 g) has a total weight of 850 g [27] and covers seven modules. The expected loading of each module is therefore calculated as 121 g. Based on Figure 3, measurement error is found to be 0.3 g in this loading range, and therefore for a whole plate would be maximal 2.1 g (0.25 percent). However, as Figure 6 demonstrates, due to the high spatial resolution of the measurement instrument, we can locate where the changes in weight are taking place on a plate. Taking a single bite is likely to cause a response from one to a maximum of three modules. To this end, the measurement error for a single bite is therefore likely to be significantly smaller than 2.1 g. Individual bites could be well identifiable in such a scenario. Mertes et al. [28] developed an instrumented dining plate that is able to measure and locate bites on a dining plate. The measurement error that was reported in this study ranged between 0.1–9.6 percent, depending on the weight that was put on the plate and the conditions in which the plate was tested (controlled lab setting vs. at-home setting).

### 4.3. Spatial Representation of Dining Interactions

Social dining defines itself by many actions that take place on the same surface, potentially even simultaneously. Insights into the spatial localisation of the interactions on the table are necessary to classify events correctly. A high spatial resolution of the measurement surface is therefore an essential element for this. That is, when the spatial resolution of a measurement instrument is low, such as the dining table by Chang et al. [25], one cannot distinguish between picking food from the dining plate or a serving tray when they are positioned above the same measurement unit. Figure 5 and Figure 6 illustrate the advantage of having high spatial resolution in terms of item localization. Moreover, Figure 7 illustrates the added value of high spatial resolution in terms of visualizing the overt and covert social aspects of eating around a table.

### 4.4. Classification of Dining Related Events

The current level of maturity of our event classification algorithm is an important focus point. This process is still ongoing, and involves fusion of data at many levels of the system: from a single module, to the superposition of multiple modules, to the surface of the entire table. Ideally, the system should evolve into a measurement instrument upon which dining plates are automatically detected, even if they change location throughout a meal, where the resting of elbows at the table is classified as non-food related behavior, and passing a water carafe along multiple table members and putting it down with a different weight and a different location, is classified correctly. This not only requires that the system is able to classify events at the moment they occur, but also that the system interprets new data in relation to events that occurred in the past. For example, the event of cutting food in pieces is likely to occur first before the pieces are speared with a fork and taken off the plate, and the event of drinking is likely to occur after someone has poured water in his glass. The data that are shown in the study by Mattfeld et al. [29] illustrates the difficulty that lies in event classification when recording social dining actions under free-living conditions. Here, one load cell was positioned under a dining tray, on which all actions took place. In their setup, they compared restricted eating in a laboratory setting, which is similar to our setup, and unrestricted eating in a cafeteria environment. The data shows that measuring eating in less controlled environment introduces noise which causes difficulties in identifying such things as bite times, bite weights, and periods of chewing. This data emphasizes the importance of linking different characteristics and events to each other. It is an essential step in making our system smart.

### 4.5. Future Work

For future work, a specific focus is the current need to manually classify which food item was placed at which position of the table, at the start of the meal. The system is only able to detect changes in weight distribution over time and does not automatically link this data to the type of food it concerns. Currently, when a certain amount of food is taken from a central plate, and the same amount of food appears at a specific location on a dining plate, one could retrospectively identify the type of food that is taken by each individual. However, one can imagine complex scenarios where multiple table members simultaneously take similar amounts of food from different serving plates, and position them on their individual dining plate. In such scenarios, event classification alone might not be enough to keep track of the food throughout the meal. Chang et al. [25] uses RFID tags (on serving plates) and RFID readers (on the table surface) in their experimental set-up, to identify each food item on the table. This set-up requires the placement of the RFID labeled dining plates, serving trays, pots, and glasses at fixed positions at the table, in order to be able to track them throughout a meal. Consequently, this limits the natural setting of the experiment and potentially the natural behavior of the diners. Ofei et al. [30] and Zhou et al. [12] show examples where the dimension of food weight and food type are merged by combining weighing scales and/or video cameras. Albeit the use of video cameras could also expose other valuable dimensions of social dining, such as facial expressions, gesture mimicking, or conversation [21,22,23,24], a disadvantage of such a set-up is the need to merge both information streams offline, rather than in real time. This might limit the potential of the system to serve as an instrument that could investigate the continuous cycle of feedback and the response to this feedback in real time. Depending on the individual health goals of the people sitting at the table, this information might be of significance to the system and play a crucial role in giving feedback—for instance, when providing feedback to the elderly with a low-carb diet, such as for diabetics.

The configuration of the hardware in our system allows for the addition of different sensors to the measurement set-up. Currently, all 199 modules are composed of a loadcell and a LED panel, but, for future purposes, it might be of added value to replace a few modules that are positioned at strategic places at the table with different sensors that could compensate for the current limitations discussed.
